# Augmenting yogurt quality attributes through hydrocolloidal gums

**DOI:** 10.5713/ajas.18.0218

**Published:** 2019-10-26

**Authors:** Lubna Rafiq, Tahir Zahoor, Ambreen Sagheer, Nazia Khalid, Ubaid ur Rahman, Atif Liaqat

**Affiliations:** 1 National Institute of Food Science and Technology, University of Agriculture Faisalabad, Faisalabad 38000, Pakistan; 2 Lahore College of Home Economics, Gulberg Lahore 54000, Pakistan; 3 School of Food and Agricultural Sciences, University of Management and Technology, Lahore 54770, Pakistan; 4 Department of Food Science and Technology, Khwaja Fareed University of Engineering and Information Technology, Rahim Yar Khan 64200, Pakistan

**Keywords:** Yogurt, Syneresis, Viscosity, Xanthan Gum, Guar Gum

## Abstract

**Objective:**

The present work was undertaken to determine the possibility of using xanthan and guar gums as stabilizers to enhance the yogurt quality.

**Methods:**

Yogurt was manufactured from standardized milk (3.5% fat, 8.5% solid-not-fat contents) with the addition of 2% to 3% starter culture. Enzyme-hydrolyzed xanthan gum (0.1%, 0.5%, 1.0%) and guar gum (0.1%, 0.5%, 1.0%) were added to the yogurt as stabilizers. Prepared yogurt samples were kept at refrigeration temperature (4°C±2°C) for 21 days and various quality and sensory parameters were studied at regular intervals (7 days).

**Results:**

Results showed that yogurt with 0.5% xanthan gum (T5) was best in terms of preventing syneresis and improving the viscosity, water holding capacity and texture of the product. Additionally, adding gums did not adversely affect the sensorial attributes of the product.

**Conclusion:**

Modified gums were found useful in augmenting yogurt quality and therefore addition of gums is highly recommended for manufacturing yogurt.

## INTRODUCTION

Fermented foods have been extensively consumed around the globe because of their nutritional importance and improved sensory attributes. Traditionally available fermented products in the market include yogurt, cheese, kefir, nonalcoholic and alcoholic beverages, several types of breads and other bakery products, vinegar and different fermented vegetables [1]. Fermented milk products are claimed to have high vitamin and mineral contents along with reduced fat contents and offer tremendous potential for promoting health and reducing the risks of various lifestyle-related ailments [2].

Yogurt is considered as the most common fermented dairy product and has been con sumed by a large group of people as a part of diet or refreshing drink. It is a multi-faceted colloidal arrangement developed due to the binding of water molecules with prolonged tiny structures of proteins [3]. Yogurt also contains significant amount of proteins having high biological value, traces of mono- and disaccharides and appreciable quantities of minerals such as sodium, potassium, calcium and magnesium etc. Besides, it also possesses considerable quantities of several other health promoting substances such as vitamin A, biotin, thiamine, riboflavin, folic acid, nicotinic acid, pantothenic acid and ascorbic acid [4]. Additionally, yogurt also has several therapeutic impacts such as enhancing the digestion & immune functionality and reducing the serum cholesterol level [5].

Globally, the demand for different types of yogurts has been increased due to more concern about product quality and consumer’s satisfaction. This augmented demand for yogurt consumption has been ascribed to improved knowledge regarding the health assistances of yogurt, growing availability of fruit and flavored yogurts and the diverseness of product presentations. Furthermore, yogurt is thought to be healthy due to its probiotic effect. Yogurt bacteria are known as probiotics and possess various health-promoting characteristics including prevention from gastrointestinal disorders, enhancement of lactose digestion by mal-digesters, mitigation of cancer risks, lowering blood cholesterol levels, strengthening the immune system and aiding the body in protein, calcium and iron assimilation [6].

Viscosity of the yogurt is generally influenced by homogeni zation process, heat treatment and yogurt processing conditions whereas syneresis usually onsets due to several factors such as high incubation temperature, low solid contents in the milk, excessive whey protein to casein ratio and physical mishandling of the product during processing, storage and transportation. The two major problems associated with yogurt are changes in the viscosity and leakage of whey proteins i.e., syneresis both of which negatively affect the yogurt quality. To overcome these glitches and to enhance the product functionality, the most common approach is the use of different stabilizers, ingredients added to the food products for smoothening and providing the uniform structure to the product. Additionally, stabilizers are also helpful in keeping the flavoring compounds in the dispersed form resulting in the maintenance of yogurt viscosity. Stabilizers also make strong networks with casein molecules which ultimately reduce the problem of syneresis and improve yogurt texture [7].

Different types of stabilizers like starch, gelatin, pectin and hydrocolloidal gums (e.g. xanthan gum and guar gum) are used to enhance the quality of yogurt. The prime focus of incorporating stabilizers in the yogurt is to improve its characteristics such as appearance, stability, viscosity, mouthfeel and texture. Additionally, sensorial attributes of yogurt are also positively affected by adding different stabilizers. Amongst various types of stabilizers, gums are thought to be most appropriate because of their high gelation properties. Commonly used gums as stabilizers include guar gum, xanthan gum, carrageenan, locust bean gum, gum acacia, konjac and tara gum [8]. Among these, natural gums viz. xanthan and guar gum are quite valuable because these are safe as compared to the synthetic stabilizers.

Several investigations have confirmed the potential of xan than gum and guar gum as stabilizers to improve yogurt stability and minimize the problem of syneresis [9]. For instance, xanthan gum is useful to improve the chemical, rheological, structural and sensory properties of yogurt [10]. Moreover, the problem of syneresis was also controlled during storage of samples containing different concentrations of gums. In addition, samples with added stabilizers also showed higher sensory scores compared to the other treatments. Likewise, guar gum is also reported to prevent syneresis and improve the texture of dairy products [11]. Besides, addition of partially hydrolyzed guar gum in low fat yogurt is helpful in reducing the whey separation and enhancing the textural and rheological properties of yogurt [12]. The above-mentioned work has proven the competence of xanthan gum and guar gum as potential stabilizers to improve the quality of yogurt. Therefore, it is necessary to introduce these stabilizers at commercial level to provide stability to yogurt and other dairy products. Moreover, the most commonly used stabilizers in the dairy industry are synthetic in nature and may impose adverse effects on human health. Consequently, these synthetic food additives should be replaced with natural ones. Accordingly, the current project was designed with the aim to evaluate the capability of hydrolyzed gums to increase yogurt stability and assess the impact of these gums on sensory attributes of yogurt. Moreover, in most of the previous studies gums were used without hydrolysis which usually causes the problem of phase separation in dairy products due to interaction between proteins and polysaccharides but in this study, gums were used after hydrolysis which is useful in controlling the problem of phase separation because hydrolysis reduces the chain length of polysaccharides.

## MATERIALS AND METHODS

The research work was conducted in Food Microbiology and Biotechnology Laboratory of National Institute of Food Science and Technology (NIFSAT), University of Agriculture, Faisalabad-Pakistan. Details of the work are provided in this section.

### Procurement of raw material

Standardized pasteurized milk was procured from Nestle Pakistan (Pvt.) Ltd. Modified gums (xanthan and guar gum) and other chemicals needed to conduct various analyses were purchased from Sigma Aldrich (Darmstadt, Germany), Oxoid (Basingstoke, UK), and Merck (Darmstadt, Germany).

### Experiment plan for gum addition and yogurt manufacturing

**Figure 1:**
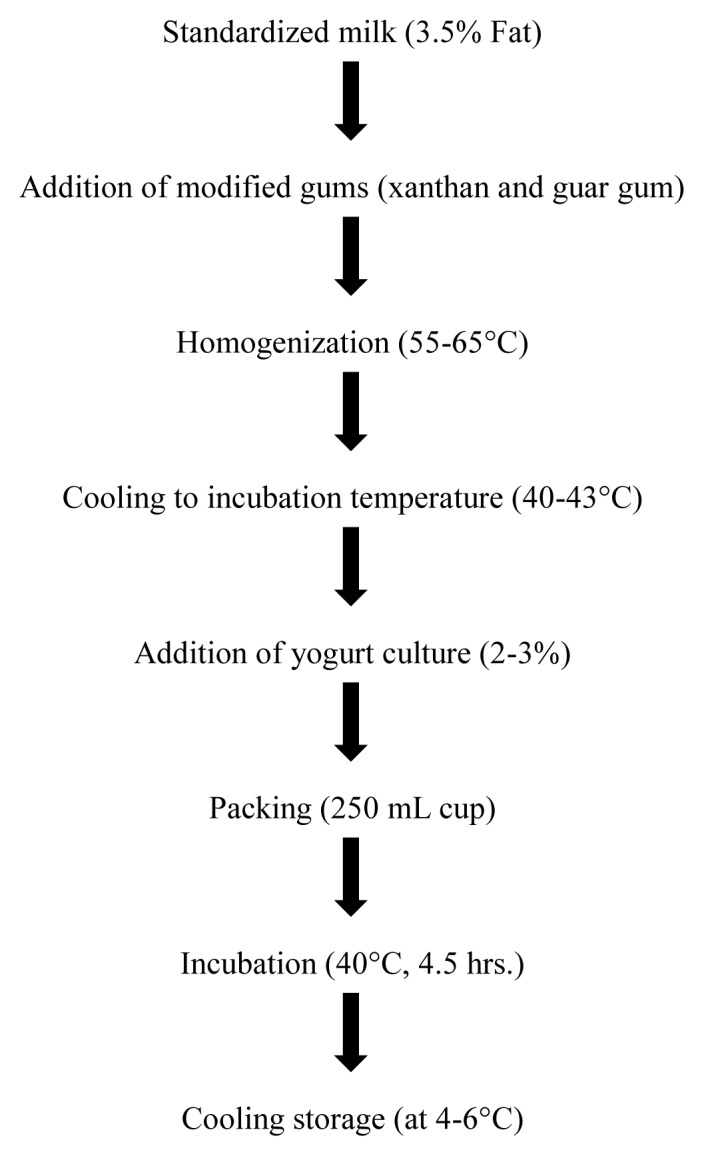
Flow diagram of yogurt preparation.

Xanthan gum and guar gum were added in different concentrations as stabilizers followed by yogurt preparation according to the flow diagram illustrated in Figure 1. The samples were grouped into 7 classes depending on the percentages of gums added during yoghurt preparation i.e., T_0_ = control (without gum addition); T_1_ = 0.1% enzyme hydrolyzed xanthan gum (EHXG); T_2_ = 0.5% EHXG; T_3_ = 1.0% EHXG; T_4_ = 0.1% enzyme hydrolyzed guar gum (EHGG); T_5_ = 0.5% EHGG; T_6_ = 1.0% EHGG. Yoghurt was prepared from standardized milk (3.5% fat) by adding 2.5% of starter culture (freeze-dried commercial culture, YO-MIXTM 300, LYO 100 DCU, Danisco, Sassenage, France) containing *Lactobacillus bulgaricus* and *Streptococcus thermophilus*. Pre-culture was prepared by dissolving 10 mg of freeze-dried culture in 50 mL milk followed by activation at 40°C for 30 minutes. Afterwards, the prepared cultured was used in yoghurt preparation at 2.5%.

### Quality analyses of yogurt

The prepared yogurt was subjected to the following analyses on weekly basis for 21 days.

### Viscosity determination

The viscosity of yogurt was estimated by using Brookfield LVDVE-230 (Cole-Parmer Scientific Experts, East Bunker Ct Vernon Hills, IL, USA) viscometer. Before viscosity determination, yogurt was stirred for 40 seconds. Afterword, viscosity was measured with the viscometer at 15°C±1°C using spindle number 4 (10 rpm). Viscometer reading was noted in centipoises (CPS) units and percent torque.

### Syneresis

The whey released by the yogurt samples was analyzed by taking 5 mL of yogurt followed by centrifugation at 5,000 rpm for 20 min at 4°C and separated whey was measured after 1 min. Amount of whey separation was expressed as volume of separated whey per 100 mL of yogurt.

### pH measurement

Digital pH meter was used for pH determination of yogurt samples according to the method given in AOAC [13]. pH meter was firstly calibrated using standard buffer solutions followed by pH measurement of samples by dipping the electrodes in the sample.

### Titratable acidity

Acidity was determined by direct titration method described by AOAC [13]. For this purpose, homogenized yogurt sample (9 mL) was taken in a beaker followed by addition of 1 to 2 drops of phenolphthalein indicator. After that titration was performed against N/10 NaOH until a slight pink color appeared as the end point. The percent acidity (as lactic acid) was calculated by using the following expression.

$$Acidity\;(\%)=\frac{0.009\times 0.1\;\text{N NaOH }(\text{mL})}{Wt.of\;sample\;(g)}\times 100$$

### Total solid contents

Total solid contents (TSCs) were determined by following the protocol described in AOAC [13]. Purposely, 5 g sample was taken in a clean dried china dish (weighed). After that the sample was subjected to heat treatment in a water bath for 15 min. and then kept in a hot air oven for 3 h at 100°C followed by cooling in a desiccator for half an hour and weighing. Percentage of TSCs was calculated by using following equation.

$$TSC\;(\%)=\frac{Wt.of\;residues}{Wt.of\;sample}\times 100$$

### Determination of water holding capacity

Water holding capacity (WHC) was determined by the method as described by Alvarez-Sabatel and coworkers [14]. Twenty grams yogurt was centrifuged for 10 min at 669×g and 20°C in a model 3K-30 laboratory centrifuge (Sigma, Darmstadt, Germany). The whey expelled was recovered and weighed. The WHC was determined by using following formula.

$$WHC\;(\%)=\frac{Wt.of\;sample\;before\;centrifugation-Wt.after\;centrifugation}{Wt.of\;sample\;before\;centrifugation}\times 100$$

### Textural analysis

Textural analysis was performed on texture analyzer (Stable Micro Systems, Godalming, Surrey, UK) using back extrusion plate Probe P-75 (75 mm Dia.) [15]. Texture Exponent 32 software was used to run the texture analyzer. The compression was done within the container at test speed of 0.5 mm/s, holding time for 2 s and 200 cps rate for data acquisition. Firmness, consistency, cohesiveness and adhesiveness of yogurt were determined to measure complete textural profile.

### Descriptive sensory analysis

The sensory evaluation of prepared yogurt samples was carried out by using 9-point hedonic scale (9 = like extremely; 1 = dislike extremely) at different storage intervals [16]. Accordingly, descriptive sensory response for various quality traits of yogurt like appearance, flavor, mouthfeel, color, texture and overall acceptability were recorded. All the evaluations were conducted by the panelists in separate booths under clear white fluorescent light in the Sensory Evaluation Laboratory of NIFSAT, University of Agriculture, Faisalabad. During evaluation process, they were provided unsalted crackers, mineral water and expectorant cups to neutralize and rinse their taste receptors for rational assessment. The descriptors were rated using a scale, with “0” as the least score for the descriptor and “9” as the highest for the descriptor. Treatments rated above “5” were considered as acceptable by descriptors. The panelists were requested to rate the product quality by scoring for the selected parameters.

### Statistical analysis

The obtained data were subjected to statistical analysis using completely randomized design (CRD) under 2-factor factorial arrangement [17]. All statistical analyses were performed using software Statistic 8.1.

## RESULTS

### Determination of yogurt viscosity

Results regarding the viscosity determination of yogurt samples containing varied amounts of modified gums are presented in Table 1. The statistical analysis revealed that treatments and storage interval significantly affected the viscosity of yogurt samples. At initiation of the storage (Day 0), means for viscosity among different treatments varied from 2,173.8±0.64 to 3,700.0 ±112.84 cps whereas at termination (Day 21), means ranged from 1,167.0±53.6 to 1,638.7±67.50 cps. The decrease in the values of viscosity was due to the development of syneresis during the storage period. It was also observed from the findings that highest mean value for viscosity was attained by T_5_ (0.5% EHGG) during storage (2,844.5 cps) followed by T_6_ (1.0% EHGG) i.e. 2,710.5 cps, while the lowest value was recorded for T_0_ (control) i.e. 1,721.6 cps.

### Syneresis

**Figure 2:**
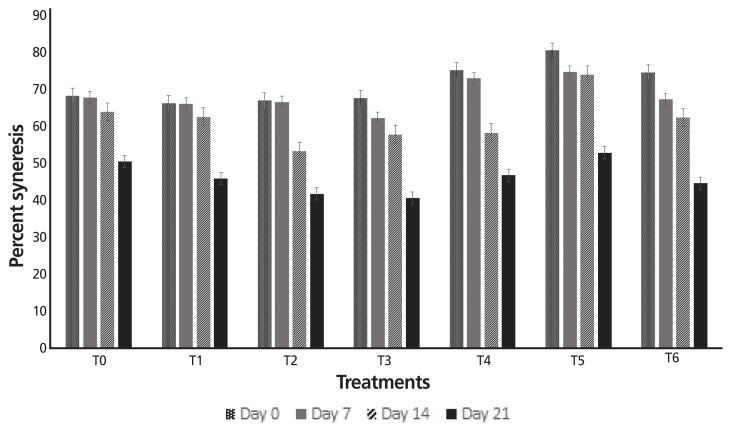
Percent syneresis values of yogurt containing modified gums during storage period. T_0_, control (without gum); T_1_, 0.1% EHXG; T_3_, 1.0% EHXG; T_2_, 0.5% EHXG; T_4_, 0.1% EHGG; T_5_, 0.5% EHGG; T_6_, 1.0% EHGG. EHXG, enzyme hydrolyzed xanthan gum; EHGG, enzyme hydrolyzed guar gum.

It is evident from the inferences regarding syneresis of yogurt that the syneresis percentages varied in a significant manner due to treatments and storage time. Among all the treatments, an increasing trend in the syneresis percentages was observed from Day 0 (38%) to the 21th day of storage (71.42%) (Figure 2). The results also showed that T_2_ (0.5% EHXG) secured minimum increase in syneresis percentage i.e. 38% to 62.42% followed by T_5_ (0.5% EHGG) i.e. 38% to 63% during the storage kinetics.

### Determination of pH and titratable acidity

**Figure 3:**
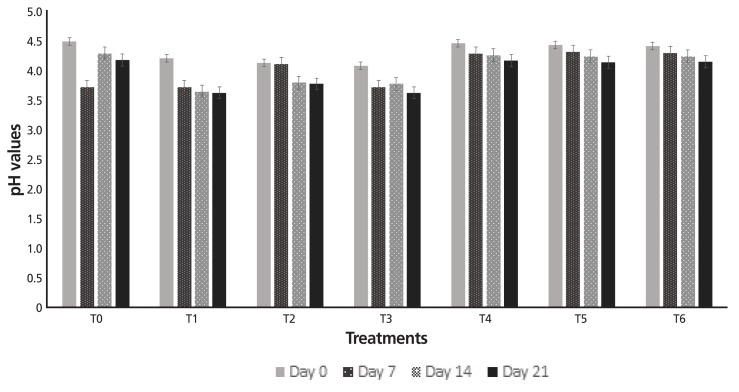
pH values of yogurt containing modified gums during storage. T_0_, control (without gum); T_1_, 0.1% EHXG; T_2_, 0.5% EHXG; T_3_, 1.0% EHXG; T_4_, 0.1% EHGG; T_5_, 0.5% EHGG; T_6_, 1.0% EHGG. EHXG, enzyme hydrolyzed xanthan gum; EHGG, enzyme hydrolyzed guar gum.

**Figure 4:**
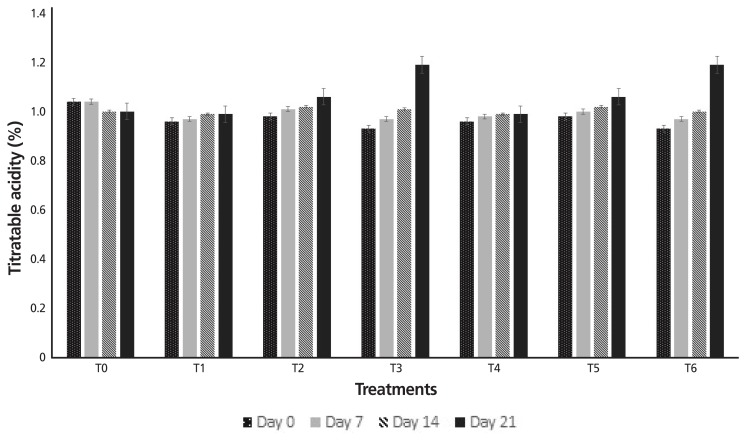
Acidity values of yogurt containing modified gums during storage. T_0_, control (without gum); T_1_, 0.1% EHXG; T_2_, 0.5% EHXG; T_3_, 1.0% EHXG; T_4_, 0.1% EHGG; T_5_, 0.5% EHGG; T_6_, 1.0% EHGG. EHXG, enzyme hydrolyzed xanthan gum; EHGG, enzyme hydrolyzed guar gum.

Figure 3 and Figure 4 illustrate the results of pH and acidity analysis of yogurt prepared by adding different percentages of hydrolyzed gums. Statistical analysis regarding pH and acidity revealed that treatments and storage period significantly affected the both parameters. The highest mean pH value was observed by T_5_ (0.5% EHGG) i.e. 4.33, trailed by T_4_ (0.1% EHGG) i.e. 4.32 whereas the lowest mean pH value (3.82) was recorded in T_1_ (0.1% EHXG) and T_3_ (1.0% EHXG) collectively. Additionally, a decreasing pattern was observed in pH values (4.34 to 3.98) during the storage period of 21 days.

The results pertaining acidity analysis of yogurt containing modified gums are also depicted in Figure 3. The data exposed that treatments, storage time and their interactive effect was significant. The statistical analysis explained that acidity values of all the yogurt samples containing stabilizers were increased during the storage period. Highest acidity value (1.02%) was recorded by T_3_ (1.0% EHXG) and T_6_ (1.0% EHGG) while the lowest acidity value (0.98%) was acquired by T_1_ (0.1% EHXG) and T_4_ (0.1% EHGG).

### Total solid contents of yogurt containing modified gums

Table 2 delineates the statistical results of TSCs of yogurt having different levels of xanthan and guar gums. The TSCs varied from 12.36% to 13.70% among the treatments at Day 0 and changed from 7.03% to 11.97% at Day 21. The highest mean value for TSCs was observed in T_5_ (0.5% EHGG) i.e. 11.97% followed by 11.96% in T_6_ (1.0% EHGG) at the end of storage period.

### Water holding capacity

The statistical data concerning WHC of yogurt samples indicated that WHC decreased during storage due to increase in acidity and syneresis. The results in Table 3 show that T_5_ (0.5% EHGG) had the maximum value for WHC (79.80%) at the start of the trial and remained highest at the termination of storage time (52.40%).

### Textural profiling of yogurt with added gums

Data concerning the textural profile of yogurt containing modified gums revealed that both factors (treatments+storage time) imposed a significant effect on the textural attributes of yogurt (Table 4). Results revealed that firmness was increased positively due to the addition of stabilizers. Highest firmness value (0.97) was recorded for T_5_ (0.5% EHGG) and T_6_ (1.0% EHGG) whereas lowest value (0.66) was observed in T_0_ (control).

Results regarding the variations in consistency of the stored yogurt are also presented in Table 4 and the mean values described that consistency was greatly affected by a storage period of 21 days. Highest mean value of consistency was 38.97 in T_5_ (0.5% EHGG) followed by T_2_ (0.5% EHXG) i.e. 28.27. On the other hand, the lowest consistency value (17.82) was attained by T_0_ (control). Similarly, cohesiveness results also showed that variations were highly significant among treatments and storage days. Highest mean value of cohesiveness was −0.40 in T_5_ (0.5% EHGG) trailed by T_6_ (1.0% EHGG) i.e. −0.39 and the lowest value (−0.26) was shown by T_0_ (control) and T_1_ (0.1% EHXG). Data regarding adhesiveness values depicted that highest retention of adhesiveness (3.14) was found for T_5_ (0.5% EHGG) followed by T_4_ (0.1% EHGG) whereas lowest value (1.89) was documented for T_0_ (control).

### Descriptive sensory analysis

Results regarding sensory parameters viz. appearance, flavor, body and texture mouth feel and overall acceptability, of yogurt samples are shown in Table 5. Variations in appearance of the product during storage period were found to be significant. Appearance score decreased from 7.40 at 0 day to 6.35 at 21st day of storage. The overall treatment means for sensory scores among all treatments were observed; 7.45 in T_0_ (control), 7.37 in T_1_ (0.1% EHXG), 7.33 in T_5_ (0.5% EHGG), 6.66 in T_2_ (0.5% EHXG) and T_6_ (1.0% EHGG), 6.62 in T_4_ (0.1% EHGG) and 6.37 in T_3_ (1.0% EHXG). Flavor scores showed a decreasing trend during 21 days of storage and values decreased from 7.57 to 6.21 from 0 to 21st day of storage. Among the mean values of treatments, T_5_ (0.5% EHGG) had the highest score i.e. 7.33 followed by T_1_ (7.21) whereas T_3_ (1.0% EHXG) got the lowest score i.e. 6.41. Results also revealed that T_5_ (0.5% EHGG) showed better sensory scores as compared to the other treatments.

Statistical results regarding mouth feel depicted a highly significant effect of treatment and storage days on mouth feel of yogurt while the effect of their interaction (days×treatments) was non-significant. Sensory scores for mouth feel decreased during storage from 7.21 on 0 day to 6.33 on 21st day of storage. T_5_ (0.5% EHGG) had highest score for mouthfeel i.e. 7.33 followed by 7.16 in T_1_ (0.1% EHXG), and lowest value (6.37) was attained by T_3_ (1.0% EHXG). Results also illustrated that color scores decreased during the storage time from 7.57 at 0 day to 6.21 at 21st day of storage. Mean values for treatments exhibited highest color score in T_5_ (7.33) followed by 6.83 in T_6_ (1.0% EHGG) while the lowest value was 6.41 for T_3_ (1.0% EHXG). Additionally, texture scores for different treatments of yogurt also decreased during storage ranging from 7.28 (Day 0) to 6.30 (Day 21). T_5_ (0.5% EHGG) showed greatest value i.e.7.33 followed by T_1_ (7.20) whereas lowest score was obtained by T_4_ (6.50). Results also indicated that overall acceptability of the product was also affected in a significant way by stabilizers during storage. Amongst treatments, highest mean value was observed in T_5_ (7.87) followed by 7.20 in T_1_ (0.1% EHXG) whereas lowest sensory score (6.37) was recorded T_4_ (0.1% EHGG) and T_2_ (0.5% EHXG).

## DISCUSSION

Use of various stabilizers in cultured dairy products is quite important to control the problem of phase separation. Stabilizers also prevent syneresis and provide smooth mouth sensation by binding water. Some stabilizers also interact with proteins and increase hydration. In yogurt, stabilizers are generally used to increase viscosity, prevent syneresis and improve mouth-feel. Similarly, results of the present work show the role of hydrolyzed gums in improving the quality of yogurt. Findings of the current project demonstrated that viscosity was decreased with the storage period, but gum-containing yogurt showed higher viscosity as compared to the control group. The improved viscosity due to addition of stabilizers to yogurt is attributed to enhanced shear-thinning, time-dependency and viscoelasticity of the product. Polysaccharides also absorb water and swell which ultimately results in increasing the viscosity. However, a decrease in the values of viscosity with the passage of time was due to the development of syneresis during the storage period. The outcomes of the present investigation are supported by the findings of Ramasubramanian et al [18] who observed a similar trend in probiotic yogurt during the storage period. Additionally, the reduction in the viscosity of yogurt during the progression of storage time can also be explained by enzymatic activity of bacteria on the casein micelle matrix [19]. A comparatively lower decrease in the samples containing different concentrations of xanthan and guar gums may be attributed to the stabilizing effect of added gums.

Additionally, the augmented percentage s of syneresis were due to an increase in the acidity of yogurt with the passage of time which resulted in separation of whey proteins (serum). However, yogurt samples containing modified gums showed a smaller increase in syneresis percentages during the storage which can be described to the stabilizing potential of added gums. Stabilizers generally control the problem of syneresis in dairy products by binding the water molecules which reduces flow of water in the matrix space. The findings of present investigation are in accordance with the work of Galal et al [20] who demonstrated a direct relationship between syneresis and storage period.

A decreasing pattern was observed in pH values during the storage period which was attributed to the conversion of lactose into lactic acid with the passage of time. Consequences of the current project are in line with the findings of Mazloomi et al [21] who inferred the similar pH variations in stabilizer-containing yogurt during storage. The conjectures of the current work regarding acidity values of yogurt containing different levels of modified gums are in conformance with the findings of Gueimonde et al [22] who reported that acidity of the yogurt increased during the storage period due to conversion of lactose into lactic acid. Results are also in accordance with Karaca [23] who studied physicochemical and sensory attributes of probiotic yogurt manufactured by adding stabilizers and investigated that the acidity of yogurt increased with storage period. Furthermore, the results pointed out that TSCs were increased by adding modified gums to yogurt. The findings of present study are in line with the results of Penna et al [24] who postulated the same trend regarding the variations in TSCs of yogurt during storage period.

Outcomes of the present investigation also postulated that WHC values of all the yogurt samples were decreased during the storage but comparatively less reduction was noticed in samples containing gums. The current findings are supported by the results of Galal et al [20] who reported that WHC values of yogurt samples decreased due to increase in syneresis during increased storage time. Moreover, inferences of textural properties of yogurt containing modified gums explored that addition of gums and storage time effected the texture of yogurt. The increased firmness was attributed to the control of syneresis and maintenance of WHC due to added gums during the storage. The findings of present study are in accordance with Seckin and Baladura [25] who studied the stabilizing effect of gums on texture of yogurt and concluded that the firmness of yogurt increased during storage. Results also showed that modified gums have positive effect on cohesiveness of yogurt and at higher concentrations of gums i.e. 1% and 0.5%, yogurt samples retained good cohesiveness levels during storage. Similarly, findings of the current study reported that there was a steady increase in adhesiveness with the increase in gum concentration.

Results regarding sensory evaluation of yogurt samples de picted that sensory parameters viz. appearance, mouthfeel, flavor, color, texture and overall acceptability of the product were affected in a significant way by stabilizers during storage. Results showed that addition of gums as stabilizer increased the overall acceptability of yogurt. Findings of the present work are in harmony with Milani and Koocheki [26] who concluded that addition of gums as stabilizer improved the texture, flavor and overall acceptability of yogurt.

## CONCLUSION

The present work demonstrates that use of xanthan and guar gums as stabilizers is an effective approach in improving the yogurt quality by enhancing viscosity, WHC and textural profile of yogurt and combating the problem of syneresis. It was concluded from the study that EHGG at 1.0% showed best results for physicochemical, textural and sensory properties of yogurt. It was also observed that with the addition of 0.5% EHGG (T_5_), the maximum reduction in syneresis and retention of WGC of the yogurt was obtained as compared to other fractions and types of gum. These findings imply that use of hydrolyzed xanthan and guar gums could produce the yogurt with high quality and better sensory attributes. Hence, it can be concluded that use of xanthan and guar gums can be promoted at industrial level to improve the texture, firmness and viscosity of yogurt and to reduce the problem of syneresis during the marketing and storage of yogurt.

## Figures and Tables

**Table 1:** Viscosity analysis (centipoise) of yogurt containing modified gums at various storage intervals

Treatments	0	7	14	21
T_0_	2,173.8±0.64^g^	1,966.5±80.63^h^	1,780.5±73.01^i^	1,442.0l±39.6^j^
T_1_	3,155.0±129.35^c^	1,699.0±78.08^i^	1,656.8±76.21^ij^	1,442.0l±66.33^j^
T_2_	3,273.8±150.30^bc^	2,912.0±133.95^e^	2,142.5±98.55^g^	1,167.0±53.6^m^
T_3_	3,270.0±150.42^bc^	2,948.0±135.61^de^	2,520.9±115.95^f^	1,462.0±67.25^kl^
T_4_	3,414.6±86.70^b^	3,110.0±127.51^cd^	2,360.2±96.76^f^	1,620.0±66.42i–k
T_5_	3,700.0±112.84^a^	3,212.7±61.52^c^	2,826.6±115.89^e^	1,638.7±67.50^ij^
T_6_	3,610.0±148.01^a^	3,178.8±130.65^c^	2,524.4±103.50^f^	1,528.8±62.34j–l

T_0_, control (without gum); T_1_, 0.1% enzyme hydrolyzed xanthan gum (EHXG); T_2_, 0.5% enzyme hydrolyzed xanthan gum (EHXG); T_3_, 1.0% enzyme hydrolyzed xanthan gum (EHXG); T_4_, 0.1% enzyme hydrolyzed guar gum (EHGG); T_5_, 0.5% enzyme hydrolyzed guar gum (EHGG); T_6_, 1.0% enzyme hydrolyzed guar gum (EHGG).

a–m Different superscript letters show significant difference (p<0.05) among various treatments during storage.

**Table 2:** Total solid contents (%) of yogurt containing modified gums

Treatments	0	7	14	21
T_0_	12.38±0.05^cd^	12.147±0.02c–e	12.087±0.04c–e	11.94±0.03^e^
T_1_	13.70±0.56^a^	13.16±0.02^b^	7.68±0.07^g^	7.12±0.07^h^
T_2_	13.60±0.2^a^	13.08±0.02^b^	7.05±0.18^h^	7.06±0.03^h^
T_3_	13.09±0.12^b^	11.12±0.05^f^	11.12±0.17^f^	7.03±0.07^h^
T_4_	12.36±0.04c–e	12.13±0.02c–e	12.12±0.01c–e	11.95±0.02^de^
T_5_	12.40±0.07^c^	12.15±0.02c–e	12.15±0.02c–e	11.97±0.02c–e
T_6_	12.39±0.01^c^	12.13±0.01c–e	12.11±0.01c–e	11.96±0.01^de^

T_0_, control (without gum); T_1_, 0.1% enzyme hydrolyzed xanthan gum (EHXG); T_2_, 0.5% enzyme hydrolyzed xanthan gum (EHXG); T_3_, 1.0% enzyme hydrolyzed xanthan gum (EHXG); T_4_, 0.1% enzyme hydrolyzed guar gum (EHGG); T_5_, 0.5% enzyme hydrolyzed guar gum (EHGG); T_6_, 1.0% enzyme hydrolyzed guar gum (EHGG).

a–h Different superscript letters show significant difference (p<0.05) among various treatments during storage.

**Table 3:** Water holding capacity (%) of yogurt containing modified gums

Treatments	Storage period (days)			

	0	7	14	21
T_0_	67.68±2.77^c^	67.22±2.24^c^	63.35±2.6c–e	50.09±1.88^ij^
T_1_	65.68±3.02c–e	65.48±3.00c–e	62.01±5.47d–f	45.51±2.09^kl^
T_2_	66.42±3.00^cd^	66.02±3.05c–e	52.83±2.43^hi^	41.43±1.91^lm^
T_3_	67.09±3.09^c^	61.73±2.84e–g	57.28±2.63^gh^	40.29±1.85^m^
T_4_	74.50±3.05^b^	72.30±2.96^b^	57.75±2.37^fg^	46.34±1.9^jk^
T_5_	79.80±3.27^a^	74.05±3.04^b^	73.25±3.00^b^	52.40±2.15^i^
T_6_	73.90±3.03^b^	66.70±2.73^c^	61.82±2.53e–g	44.21±1.81k–m

T_0_, control (without gum); T_1_, 0.1% enzyme hydrolyzed xanthan gum (EHXG); T_2_, 0.5% enzyme hydrolyzed xanthan gum (EHXG); T_3_, 1.0% enzyme hydrolyzed xanthan gum (EHXG); T_4_, 0.1% enzyme hydrolyzed guar gum (EHGG); T_5_, 0.5% enzyme hydrolyzed guar gum (EHGG); T_6_, 1.0% enzyme hydrolyzed guar gum (EHGG).

a–m Different superscript letters show significant difference (p<0.05) among various treatments during storage.

**Table 4:** Effect of stabilizers on textural profile of yogurt

Treatments1)	Textural profile			

	Firmness	Consistency	Cohesiveness	Adhesiveness
T_0_	0.66^f^	17.82^d^	−0.26^a^	1.89^e^
T_1_	0.83^cd^	18.22^d^	−0.26^a^	2.29^d^
T_2_	0.73^e^	28.27^b^	−0.35^bc^	2.41^cd^
T_3_	0.93^ab^	20.34^cd^	−0.27^ab^	2.69^bc^
T_4_	0.87^bc^	20.34^cd^	−0.36^bc^	2.90^b^
T_5_	0.97^a^	38.97^a^	−0.40^c^	3.14^a^
T_6_	0.97^a^	22.66^c^	−0.39^c^	2.42^cd^
STE	0.045	2.85	0.02	0.16
SD	±0.12	±7.55	±0.06	±0.41

STE, standard error; SD, standard deviation.

1) T_0_, control (without gum); T_1_, 0.1% enzyme hydrolyzed xanthan gum (EHXG); T_2_, 0.5% enzyme hydrolyzed xanthan gum (EHXG); T_3_, 1.0% enzyme hydrolyzed xanthan gum (EHXG); T_4_, 0.1% Enzyme hydrolyzed guar gum (EHGG); T_5_, 0.5% enzyme hydrolyzed guar gum (EHGG); T_6_, 1.0% enzyme hydrolyzed guar gum (EHGG).

a–f Different superscript letters within the same column differ significantly (p<0.05).

**Table 5:** Sensory evaluation scores of yogurt samples containing various levels of xanthan and guar gums

Treatments1)	Appearance	Flavor	Mouthfeel	Color	Texture	Overall acceptability
T_0_	7.45±0.29^a^	7.45±0.04^a^	7.37±0.57^a^	7.62±0.58^a^	7.45±0.57^a^	7.41±0.04^b^
T_1_	7.37±0.07^a^	7.25±0.04^ab^	7.16±0.42^a^	7.2±0.44^ab^	7.2±0.54^a^	7.2±0.14^b^
T_2_	6.66±0.03^b^	6.66±0.02^c^	6.5±0.39^b^	6.75±0.58^c^	6.66±0.57^b^	6.37±0.68^c^
T_3_	6.37±0.16^b^	6.41±0.54^c^	6.37±0.68^b^	6.41±0.15^c^	6.62±0.29^b^	6.5±0.55^c^
T_4_	6.62±0.06^b^	6.66±0.57^c^	6.45±0.28^b^	6.45±0.71^c^	6.62±0.54^b^	6.37±0.43^c^
T_5_	7.33±0.07^a^	7.37±0.68^a^	7.33±0.43^b^	7.33±0.71^a^	7.33±0.43^a^	7.87±0.43^a^
T_6_	6.66±0.04^b^	6.83±0.29^bc^	6.62±0.43^b^	6.83±0.54^c^	6.5±0.72^b^	6.75±0.57^c^

1) T_0_, control (without gum); T_1_, 0.1% enzyme hydrolyzed xanthan gum (EHXG); T_2_, 0.5% enzyme hydrolyzed xanthan gum (EHXG); T_3_, 1.0% enzyme hydrolyzed xanthan gum (EHXG); T_4_, 0.1% enzyme hydrolyzed guar gum (EHGG); T_5_, 0.5% enzyme hydrolyzed guar gum (EHGG); T_6_, 1.0% enzyme hydrolyzed guar gum (EHGG).

a–c Different superscript letters within the same column differ significantly (p<0.05).
